# Peripheral Delta Opioid Receptors Mediate Formoterol Anti-allodynic Effect in a Mouse Model of Neuropathic Pain

**DOI:** 10.3389/fnmol.2019.00324

**Published:** 2020-02-14

**Authors:** Rhian Alice Ceredig, Florian Pierre, Stéphane Doridot, Unai Alduntzin, Pierre Hener, Eric Salvat, Ipek Yalcin, Claire Gaveriaux-Ruff, Michel Barrot, Dominique Massotte

**Affiliations:** ^1^Institut des Neurosciences Cellulaires et Intégratives, Centre National de la Recherche Scientifique, Université de Strasbourg, Strasbourg, France; ^2^Chronobiotron, Centre National de la Recherche Scientifique, Strasbourg, France; ^3^Centre d’Evaluation et de Traitement de la Douleur, Hôpitaux Universitaires de Strasbourg, Strasbourg, France; ^4^Institut de Génétique et de Biologie Moléculaire et Cellulaire, Centre National de la Recherche Scientifique, Université de Strasbourg, INSERM, Illkirch, France

**Keywords:** mechanical allodynia, beta-mimetics, peripheral nerve injury, cuff model, delta opioid receptor, beta adrenergic receptor

## Abstract

Neuropathic pain is a challenging condition for which current therapies often remain unsatisfactory. Chronic administration of β2 adrenergic agonists, including formoterol currently used to treat asthma and chronic obstructive pulmonary disease, alleviates mechanical allodynia in the sciatic nerve cuff model of neuropathic pain. The limited clinical data currently available also suggest that formoterol would be a suitable candidate for drug repurposing. The antiallodynic action of β2 adrenergic agonists is known to require activation of the delta-opioid (DOP) receptor but better knowledge of the molecular mechanisms involved is necessary. Using a mouse line in which DOP receptors were selectively ablated in neurons expressing Nav1.8 sodium channels (DOP cKO), we showed that these DOP peripheral receptors were necessary for the antiallodynic action of the β2 adrenergic agonist formoterol in the cuff model. Using a knock-in mouse line expressing a fluorescent version of the DOP receptor fused with the enhanced green fluorescent protein (DOPeGFP), we established in a previous study, that mechanical allodynia is associated with a smaller percentage of DOPeGFP positive small peptidergic sensory neurons in dorsal root ganglia (DRG), with a reduced density of DOPeGFP positive free nerve endings in the skin and with increased DOPeGFP expression at the cell surface. Here, we showed that the density of DOPeGFP positive free nerve endings in the skin is partially restored and no increase in DOPeGFP translocation to the plasma membrane is observed in mice in which mechanical pain is alleviated upon chronic oral administration of formoterol. This study, therefore, extends our previous results by confirming that changes in the mechanical threshold are associated with changes in peripheral DOP profile. It also highlights the common impact on DOP receptors between serotonin noradrenaline reuptake inhibitors such as duloxetine and the β2 mimetic formoterol.

## Introduction

Neuropathic pain arises from traumatic nerve injury or from a disease that affects the somatosensory system and is characterized by spontaneous pain, mechanical allodynia and/or thermal hypersensitivity (von Hehn et al., 2012). First-line treatments include antidepressants such as serotonin and noradrenaline reuptake inhibitors (SNRIs), or anticonvulsants such as gabapentinoids (Kremer et al., 2016a). In preclinical studies, activation of the β2-adrenergic receptors has been shown to be mandatory for the antiallodynic action of antidepressants (Yalcin et al., 2009; Kremer et al., 2016a, 2018) and chronic administration of several β2-adrenergic agonists such as formoterol has been successfully used to alleviate mechanical allodynia (Choucair-Jaafar et al., 2009, 2014; Yalcin et al., 2010; Jourdain and Hatakeyama, 2019). Formoterol is already routinely used in clinics to treat chronic obstructive pulmonary disease (Vanfleteren et al., 2018). Also, inhalation of β2-agonists during the perioperative period was associated with a five-fold lower risk of developing post-thoracotomy neuropathic pain whereas chronic antidepressants or chronic gabapentanoids appeared ineffective (Salvat et al., 2015). Drug repurposing could, therefore, be envisaged to treat neuropathic pain.

The mechanisms underlying the relief of mechanical allodynia are the topic of extensive research in various preclinical models but remain unclear. Interestingly, delta-opioid (DOP) receptors are essential for the antiallodynic effect of DOP agonists (Nozaki et al., 2012; Vicario et al., 2016) but also for the antiallodynic effect of chronic administration of both antidepressants (Benbouzid et al., 2008b; Yalcin et al., 2010; Ceredig et al., 2018; Kremer et al., 2018) and β2 mimetics (Yalcin et al., 2010; Choucair-Jaafar et al., 2014). More specifically, our previous work using the cuff model pointed to peripheral DOP receptors expressed in Nav1.8 positive neurons as mandatory for the antiallodynic action of DOP agonists (Gaveriaux-Ruff et al., 2011; Nozaki et al., 2012) as well as the SNRI duloxetine (Ceredig et al., 2018). Our data also revealed that changes in the expression profile of peripheral DOP receptors correlated with mechanical allodynia. Indeed, we observed decreased DOP receptor expression in unmyelinated calcitonin gene-related peptide (CGRP) positive neurons and free nerve endings in the skin and increased surface expression in the neurons still expressing the receptor in neuropathic conditions but not in mice chronically treated with duloxetine after cuff surgery (Ceredig et al., 2018). Here, we sought to determine whether the same mechanisms are triggered by chronic treatment with the β2 adrenergic agonist formoterol by identifying changes in the expression of peripheral DOP receptor using a mouse line in which peripheral DOP receptors are selectively ablated in neurons expressing the Nav1.8 sodium channel (DOP cKO; Gaveriaux-Ruff et al., 2011) and a knock-in mouse line expressing DOP receptors fused to the green fluorescent protein eGFP (DOPeGFP; Scherrer et al., 2006). Our data indicate that peripheral DOP receptors expressed in Nav1.8+ neurons were mandatory for formoterol anti-allodynic activity. DOP surface expression was lower in animals treated with chronic formoterol compared to neuropathic conditions. However, DOP receptor expression was only partially restored in nerve free endings in the skin and remained similar to the neuropathic conditions in the dorsal root ganglia (DRG) after chronic formoterol. Altogether, data suggest that antidepressants and β2 mimetics effects engage similar DOP-dependent mechanisms.

## Materials and Methods

### Animals

DOPeGFP knock-in mice expressing the DOP receptor infusion with the green fluorescent protein were generated by homologous recombination. In these animals, the eGFP cDNA preceded by a five amino acid linker (G-S-I-A-T) was introduced into the exon 3 of the DOP receptor gene, in the frame and 5’ from the stop codon (Scherrer et al., 2006). The genetic background of DOPeGFP mice was C57BL/6J:129SvPas (50%:50%). DOP-floxed (*Oprd1fl/fl*) mice were interbred with Nav1.8-Cre mice to generate conditional knockout (cKO) of DOP in primary nociceptive neurons (Nav1.8-Cre × *Oprd1fl/fl* or DOPcKO) as previously reported (Gaveriaux-Ruff et al., 2011). The genetic background of conditional DOP knock-out mice and their floxed controls was C57BL/6J:129SvPas (62.5%:37.5%). These mice were bred at the ICS animal facility, Illkirch, France, and kindly provided by Pr. Claire Gavériaux-Ruff. Adult male and female mice aged 6–20 weeks, weighing 20–32 g for females and 20–38 g for males were used. Animals from independent cohorts were distributed at best to provide groups of similar size for each gender and treatment (*n* = 88 DOPeGFP mice, *n* = 22 DOPcKO mice, *n* = 20 DOP-floxed mice). Mice were group-housed 2–5 per cage, under standard laboratory conditions (12 h dark/light cycle, lights on at 7 am) in temperature (21 ± 1°C) and humidity (55 ± 10%) controlled rooms with food and water *ad libitum*. All experiments were approved by the “Comité d’Ethique en Matière d’Expérimentation Animale de Strasbourg” [authorization number 201503041113547 *(APAFIS#300).02*] and conducted in agreement with the EU Directive 2010/63/EU for animal experiments.

### Neuropathic Pain Model

Neuropathic pain was induced by cuffing the main branch of the right sciatic nerve as previously described (Benbouzid et al., 2008c; Yalcin et al., 2014). Before surgeries, mice were anesthetized with ketamine (Vibrac, Carros, France)/xylazine (Rompun, Kiel, Germany; 100/10 mg/kg, i.p.). The common branch of the right sciatic nerve was exposed, and a cuff of PE-20 polyethylene tubing (Harvard Apparatus, Les Ulis, France) of standardized length (2 mm) was unilaterally inserted around it (Cuff group). Sham-operated animals underwent the same surgical procedure without cuff implantation (Sham group). Animals were placed on their left side in a clean home cage immediately after surgery and kept under the heat lamp until they awoke. Water and chow were placed directly in the home cage. The surgical site was checked daily during the next 3 days, and animals were monitored for signs of unusual suffering or infection with endpoints defined in agreement with the recommendations of the ethical committee.

### Assessment of Mechanical Allodynia

Mechanical allodynia was tested using von Frey filaments and results were expressed in grams as described in Yalcin et al. (2014). Briefly, calibrated von Frey filaments (Bioseb, Vitrolles, France) were applied to the plantar surface of each hind paw until they just bent, in a series of ascending forces up to the mechanical threshold. Filaments were tested five times per paw and the paw withdrawal threshold (PWT) was defined as the lower of two consecutive filaments for which three or more withdrawals out of the five trials were observed (Yalcin et al., 2014).

### Treatment Procedures

Treatment with the β2-adrenergic agonist formoterol began 4 weeks after the surgical procedure and lasted 4 weeks. Formoterol (Cat. Nr BG0369, Biotrend AG, Switzerland) was delivered by *ad libitum* access and as sole source of fluid dissolved in drinking water at a dose of 0.5 μg/ml (equivalent to 0.05 mg/kg/day) with 0.2% saccharin (Cat. Nr S1002, Sigma Aldrich, St Louis, MO, USA). Experimental groups for DOPeGFP mice included the Sham group (*n* = 36, 29 females and seven males), Cuff group (*n* = 29, 16 females and 13 males), and Formoterol group corresponding to Cuff mice treated with formoterol (*n* = 23, 14 females and nine males). Experimental groups for DOP cKO mice included Sham group (*n* = 6, two females and four males), Cuff group (*n* = 5 males), and Formoterol group corresponding to Cuff mice treated with formoterol (*n* = 11, seven females and four males). Experimental groups for control littermates *Oprd1fl/fl* (floxed mice) included Sham group (*n* = 7, five females and two males), Cuff group (*n* = 5 males), and Formoterol group corresponding to Cuff mice treated with formoterol (*n* = 8, two females and six males). Sham and Cuff groups both received control saccharin solution 0.2% in drinking water. Sham and Cuff groups were identical to those published previously in Ceredig et al. (2018).

### Tissue Preparation and Immunohistochemistry

Mice were anesthetized with ketamine (Vibrac, Carros, France)/xylazine (Rompun, Kiel, Germany; 100/10 mg/kg, i.p.) and perfused intracardially with 100 ml of ice-cold (2–4°C) 4% paraformaldehyde (PFA) in phosphate buffer 0.1 M pH 7.4 (PB). Ipsilateral (right) and contralateral (left) L4 to L6 lumbar DRG were dissected out and post-fixed for 90–120 mins at 4°C in 4% PFA in PB, cryoprotected at 4°C in 30% sucrose in PB for 24 h, embedded in OCT (Optimal Cutting Temperature medium, Thermo Fisher Scientific, Waltham, MA, USA), frozen and kept at −80°C. DRG longitudinal sections (16 μm thick) were cut with a cryostat (Microm Cryo-star HM560) and kept floating in PB.

Immunohistochemistry was performed according to standard protocols as previously described in Ceredig et al. (2018). Briefly, DRG sections were incubated for 1 h at room temperature (RT) in the blocking solution consisting of PB with 0.2% Tween 20 (PBT; Cat. Nr 85114, Thermo Fisher Scientific, Waltham, MA, USA), 3% normal goat serum (Invitrogen, Paisley, UK) and 3% donkey serum when needed (D9663 Sigma-Aldrich, St Quentin Fallavier, France). The sections were then incubated overnight at 4°C in the blocking solution with the appropriate primary antibodies: polyclonal rabbit anti-GFP (Cat. Nr A-11122, Invitrogen, dilution 1:1,000), sheep polyclonal anti-CGRP (Cat. Nr. AB 22560, Abcam, dilution 1:2,000). Three washes were performed with PBT before sections incubated for 2 h at RT in dim light with goat anti-rabbit IgG conjugated with Alexa Fluor 488 (Cat. Nr A-11012, Molecular Probes, dilution 1:2,000) and donkey anti-sheep IgG conjugated with Alexa Fluor 594 (Cat. Nr A-11016, Molecular Probes, dilution 1:2,000). Following three washes with PBT, the sections were mounted with MOWIOL (Calbiochem, Darmstadt, Germany) and 4,6-diamino-phenylindole (DAPI; Roche Diagnostic, Mannheim, Germany; 0.5 μg/ml).

Plantar skin of both hind paws (footpad and glabrous skin, 1 cm long) were fixed at 4°C in 4% PFA solution overnight, cryoprotected overnight with 30% sucrose in PB, embedded in OCT, frozen and kept at −80°C. Longitudinal cross-sections (50 μm thickness) were cut with a cryostat (MicromCryo-star HM560) and kept floating in PB. Paw tissue samples were then processed to visualize primary afferent terminals in the skin of the hind paw as previously described in Ceredig et al. (2018). Briefly, sections were treated with 0.3% H_2_O_2_, dehydrated with successive baths in ethanol then rehydrated, washed 3 times with PBS and incubated in blocking solution (PBS, 0.5% Triton X100 (PBST) with 3% normal goat serum or normal donkey serum) for 30 min at RT. After overnight incubation at 4°C in the blocking solution, the sections were incubated with the primary antibodies against anti-GFP (1:1,000) or anti-CGRP (1:2,000) antibody. The sections were then washed three times with PBST, respectively incubated for 2 h at RT with anti-rabbit or anti-goat biotinylated secondary antibody (1:400) in PBST and washed again three times with PBST before staining with Vector SG (Sk-4700, VectorLab). Samples were mounted with MOWIOL.

### Image Acquisition and Analysis

Image acquisition and analysis were performed as previously described in Ceredig et al. (2018). Briefly, images were acquired with the Leica TCS SP5 confocal microscope using a 20× dry objective (Numerical Aperture: 0.7), the 40× resolution was achieved with a digital zoom factor. Confocal acquisitions in the sequential mode (single excitation beams: 405, 488 and 568 nm) were used for marker co-localization to avoid potential crosstalk between the different fluorescence emissions. Images were acquired with the LCS (Leica) software using randomly selected sections.

The ImageJ^®^ software cell counter (approximately 15 non-adjacent sections per condition and per animal) was used to count on-screen neurons expressing a given fluorescent marker manually and blindly. Threshold was applied to fluorescence detection. Only neurons from L4-L6 DRGs with a visible nucleus were considered. Cells expressing a given marker and eGFP fluorescence were analyzed separately. During the analysis, we recorded all cross-sectional areas of cell profiles for each marker. No difference was observed in the distribution of the neuronal populations between male and female mice and data were pooled for subsequent analysis.

DOPeGFP subcellular distribution was expressed as a ratio of membrane-associated vs. cytoplasmic fluorescence densities determined as previously described (Erbs et al., 2016). Acquisitions using 63× (NA: 1.4) oil objective were performed to determine DOPeGFP subcellular distribution. Briefly, quantification of internalization was performed using the ImageJ software on 8-bit raw confocal images from neurons randomly sampled. Nuclear fluorescence was used to define the background level (no threshold was applied). Cytosolic fluorescence intensity was subtracted from whole-cell fluorescence intensity to obtain surface fluorescence intensity. Fluorescence intensity values were divided per surface unit (pixel) to obtain densities. Ratio of membrane-associated (Df memb) vs. cytoplasmic (Df cyto) fluorescence densities was calculated to normalize data across neurons examined. A value of 1.0 results from equal densities of DOPeGFP at the cell surface and in the cytoplasm.

Free nerve endings in the glabrous part of the skin were visualized using a 20× dry objective (Nikon Eclipse 80i). Counting on blinded samples was performed manually on screen using the Neurolucida software (V.10 MBF Bioscience) on at least four randomly chosen sections per animal. Density was obtained by dividing the number of afferents crossing the dermal-epidermal junction excluding secondary branching, by the total length of the section (Lauria et al., 2005).

### Statistical Analysis

Behavioral analysis of von Frey testing was performed using Statistica v12 (StatSoft, France) and Graph-Pad Prism v7 (GraphPad, San Diego, CA, USA). Changes in the PWT, as a function of post-operative time (within factor) and experimental treatment (between factor), were analyzed using two-way ANOVA with repeated measures (two-way rANOVA) analysis followed by Tukey HSD *post hoc* test. Baseline PWT in males and females were compared using a two-sample student’s *t*-test. Exact *p*-values below 0.0001 were not provided for behavioral data (Graph-Pad Prism v7, GraphPad, San Diego, CA, USA). For cross-sectional area measurements, data were pooled per treatment group for each marker (CGRP or eGFP). In all groups, cross-sectional areas were found not normally distributed (*p-*value always < 10^−8^, Shapiro–Wilk test) using R (R Core Team, 2017). Sums of Gaussian functions were therefore adapted to the relative cumulative distribution curve of cell size, using non-linear least-square curve fitting (with nls2, nlstools and pracma R packages). Data were expressed as cumulative distributions to allow direct determination of the mean and standard error by adjusting the function of repartition on the experimental points. We compared the cumulative distributions for the various groups using the non-parametric Kolmogorov–Smirnov test (R). Treatment impact in DOPeGFP+ bins was analyzed using multiple *t*-tests. Statistical analysis of DOPeGFP subcellular distribution and skin fiber was performed with one-way ANOVA followed by Tuckey HSD *post hoc* test (Graph-Pad Prism v7, GraphPad, San Diego, CA, USA). Co-localization of DOPeGFP with neuronal markers in small size neurons (<300 μm^2^) was analyzed using the non-parametric Kruskal–Wallis test followed by Dunn’s *post hoc* test.

## Results

### Chronic Formoterol Requires DOP Receptors Expressed in Nav1.8+ Neurons for Anti-allodynic Action

We previously established that mechanical allodynia is induced by cuff-implantation, developed directly after surgery and was maintained until up to 12–14 weeks (Yalcin et al., 2011). Also, DOP receptors expressed in Nav1.8+ neurons were shown to be mandatory for the anti-allodynic action of the antidepressant duloxetine in the cuff model (Ceredig et al., 2018). Since DOP receptors are also required for the anti-allodynic effect of a chronic treatment with the β2 agonist clenbuterol (Yalcin et al., 2010), we investigated whether the specific deletion of DOP receptors in primary afferents (peripheral DRG neurons) expressing Nav1.8 voltage-gated sodium channels (DOPcKO; Gaveriaux-Ruff et al., 2011) was also abolishing the antiallodynic action of a β2-adrenergic agonist.

**Figure 1:**
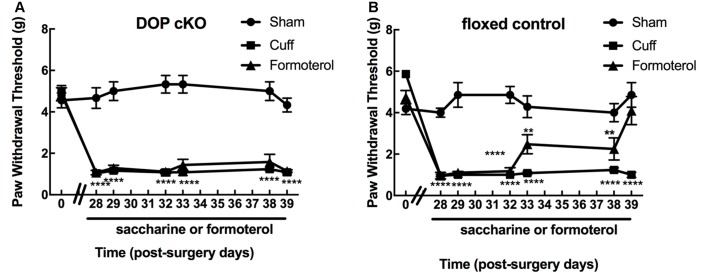
Expression of delta-opioid (DOP) receptors in Nav1.8+ neurons was mandatory for oral formoterol anti-allodynic action. The right (ipsilateral) hind paw mechanical threshold was tested using von Frey calibrated filaments. Following cuff implantation surgery, animals had lowered paw withdrawal thresholds (PWT), displaying sustained mechanical allodynia. Formoterol (0.5 μg/ml) or saccharin 0.2% control *per* os treatments started 4 weeks after nerve injury and were maintained for 3 weeks. **(A)** Mechanical threshold of the right (ipsilateral) hind paw in DOP cKO mice. Data from three separate experiments (each including the three conditions) were pooled and are expressed as means ± SEM. Sham (*n* = 6), Cuff (*n* = 5), Cuff treated with formoterol (*n* = 11). Two-way rANOVA and Tukey HSD *post hoc* test: ****p < 0.001 formoterol treatment vs. baseline. **(B)** Mechanical threshold of the right (ipsilateral) hind paw in control floxed mice. Data from three separate experiments (each including the three conditions) were pooled and are expressed as means ± SEM. Sham (*n* = 7), Cuff (*n* = 5), Cuff treated with formoterol (*n* = 8). Two-way rANOVA and Tukey HSD *post hoc* test: ****p < 0.0001, **p < 0.01 formoterol treatment day 39 vs. baseline: *p* = 0.3284.

Von Frey testing revealed that sciatic nerve cuffing resulted in unilateral mechanical allodynia in DOPcKO and control floxed (DOP*fl/fl)* animals (Figures 1A,B) as previously reported (Ceredig et al., 2018). Cuff DOPcKO mice did not recover after formoterol treatment in drinking water (50 μg/ml; two-way rANOVA, interaction treatment × time *F*_(12,114)_ = 11.31, *p* < 0.0001, effect of time *F*_(6,114)_ = 33.37, *p* < 0.0001, effect of treatment *F*_(2,19)_ = 112.8, *p* < 0.0001, Tukey HSD *post hoc* test: formoterol treatment from day 28 to 39 vs. Sham, *p* < 0.0001 at each time-point; Figure 1A), whereas control DOP*fl/fl* Cuff animals treated with formoterol returned to baseline values at day 39, after 11 days of formoterol administration (two-way rANOVA interaction treatment × time *F*_(12,119)_ = 8.386, *p* < 0.0001, effect of time *F*_(6,119)_ = 18.56, *p* < 0.0001, effect of treatment *F*_(2,119)_ = 84.47, *p* < 0.0001, Tukey HSD *post hoc* test: formoterol treatment vs. Sham: *p* < 0.0001 from day 28 to 32, *p* < 0.01 from day 33 to day 38, *p* = 0.33 on day 39; Figure 1B). Our result therefore established that DOP receptors in Nav1.8 positive neurons were mandatory to alleviate mechanical allodynia upon chronic treatment with the β2 mimetic formoterol. We thus assessed in more detail the impact of chronic formoterol treatment on neuronal populations expressing the DOP receptor using the DOPeGFP fluorescent knock-in mouse line.

### Chronic Formoterol Alleviates Cuff-Induced Mechanical Allodynia in DOPeGFP Mice

**Figure 2:**
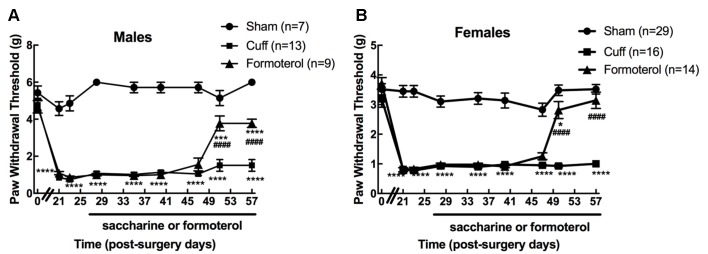
Chronic formoterol treatment *per os* relieved mechanical allodynia in DOPeGFP knock-in mice. The right (ipsilateral) hind paw mechanical threshold was tested using von Frey calibrated filaments in male and female DOPeGFP KI mice. Males **(A)** and females **(B)** had lowered PWT following cuff implantation surgery, i.e., sustained mechanical allodynia. Four weeks after nerve injury, formoterol (0.5 μg/ml) or saccharin 0.2% control *per* os treatments started and were maintained for 4 weeks. Data from three separate experiments (each including the three conditions) were pooled and are expressed as means ± SEM. Sham (*n* = 7 males and 29 females), Cuff (*n* = 13 males and 16 females), Cuff treated with formoterol (*n* = 9 males and 14 females). Two-way rANOVA and Tukey HSD *post hoc* test: *p < 0.05, ***p < 0.001, ****p < 0.0001 vs. Sham. ^####^ *p* < 0.001 vs. Cuff.

We first verified that oral administration of the β2 adrenergic agonist-induced the expected anti-allodynic effect in the DOPeGFP knock-in mouse line. As previously reported (Ceredig et al., 2018), males had significantly higher baseline mechanical thresholds compared to females (5.4 ± 0.4 g for males vs. 3.5 ± 0.2 g for females, student’s *t*-test for baseline values: *t* = 7.18 *p* < 0.0001) and cuff implantation induced ipsilateral mechanical allodynia which lasted for at least 8 weeks (time of perfusion) in either sex (two-way rANOVA; males interaction treatment × time *F*_(16,208)_ = 15.15, *p* < 0.0001; effect of treatment, *F*_(2,26)_ = 318.5, *p* < 0.0001 from day 21 to 57 Cuff vs. Sham; females interaction treatment × time *F*_(16,448)_ = 11.02, *p* < 0.0001, effect of treatment *F*_(2,56)_ = 236, *p* < 0.0001 from day 21 to 57 Cuff vs. Sham; Figures 2A,B). We did not detect any change in the nociceptive threshold after sham surgery or in the contralateral hind paw of Cuff animals (data not shown).

Formoterol treatment in drinking water (50 μg/ml) relieved mechanical allodynia at day 51 (following 23 days administration) in DOPeGFP males although not fully (two-way rANOVA Tukey HSD *post hoc* test: day 51 vs. Sham: *p*_(Males)_ = 0.0009 and day 57 vs. Sham: *p*_(Males)_ < 0.0001, day 51 and day 57 vs. Cuff: *p*_(Males)_ < 0.0001; Figure 2A). In DOPeGFP females, formoterol treatment-induced almost complete relief at day 51 (two-way rANOVA Tukey HSD *post hoc* test: day 51 vs. Sham: *p*_(Females)_ = 0.0382) and values were similar to sham mice at day 57 (two-way rANOVA Tukey HSD *post hoc* test: day 57 vs. Sham: *p*_(Females)_ = 0.3547, day 51 and day 57 vs. Cuff: *p*_(Females)_ < 0.0001; Figure 2B). This time course was similar to what we previously observed, in the same model, in male C57BL6/J mice repeatedly injected i.p. twice daily (Yalcin et al., 2010).

### DOPeGFP Expression in Formoterol-Treated Mice

**Figure 3:**
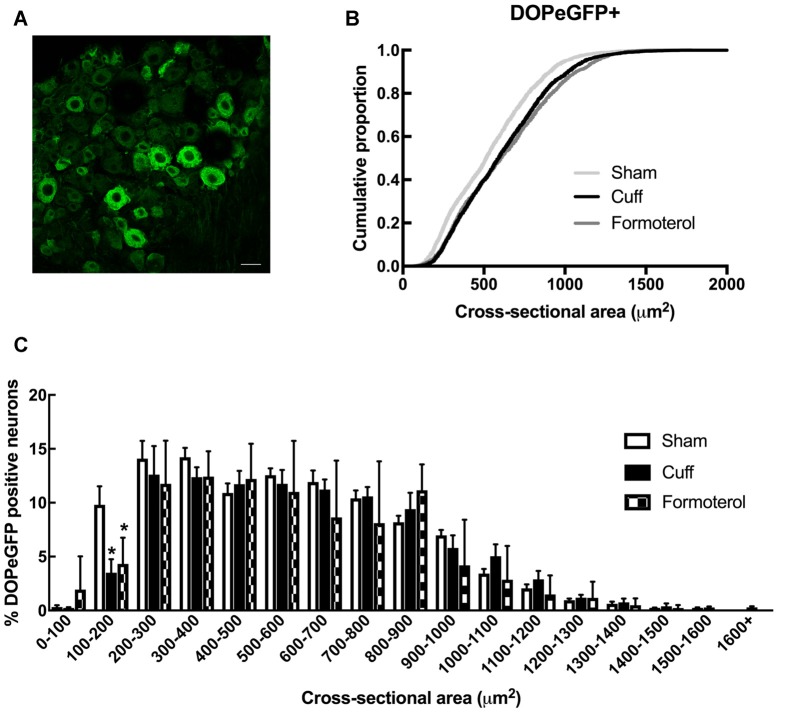
DOPeGFP distribution in Sham, Cuff and Formoterol-treated animals. **(A)** Representative confocal image of fluorescent DOPeGFP expressing neurons in lumbar dorsal root ganglia (DRG) of sham animals. Scale bar 10 μm. **(B)** Distribution of DOPeGFP+ neuronal populations in Sham (3,080 neurons, *n* = 7 animals; light gray), Cuff (3,123 neurons, *n* = 6 animals; black) and Formoterol (1,917 neurons, *n* = 5 animals; dark gray) groups. **(C)** Categorical data plot of the size distribution for DOPeGFP positive neuron cross-sectional areas in Sham (white bars), Cuff (black bars) and Formoterol (checked bars) groups. Multiple *t*-tests: *p = 0.03 Cuff vs. Sham, *p = 0.01 Formoterol vs. Sham.

We then examined changes in the distribution of DOPeGFP+ neurons in the DRG (Figure 3A). As previously established (Ceredig et al., 2018), the cumulative distribution of DOPeGFP+ cells in the Cuff experimental groups was shifted towards larger cell size values compared to the Sham group (Figure 3B). The loss of DOPeGFP+ neurons with small cross-sectional areas (Multiple *t*-tests: Cuff vs. Sham: *p* = 0.03 for the 100–200 μm^2^ category) indicated a loss of DOPeGFP expression in small and/or medium neurons following 8 weeks of neuropathy (Figure 3C).

In Cuff mice chronically treated with formoterol, the distribution of the DOPeGFP+ neuronal populations remained very similar to the Cuff condition and was significantly different from the Sham group (KS test: *D* = 0.14212, *p* < 2.2 10^−16^; Figure 3B). Indeed, the percentage of small size neurons remained lower compared to the Sham condition (Multiple *t*-tests: Formoterol vs. Sham: *p* = 0.0137 for the 100–200 μm^2^ category; Figure 3C).

**Figure 4:**
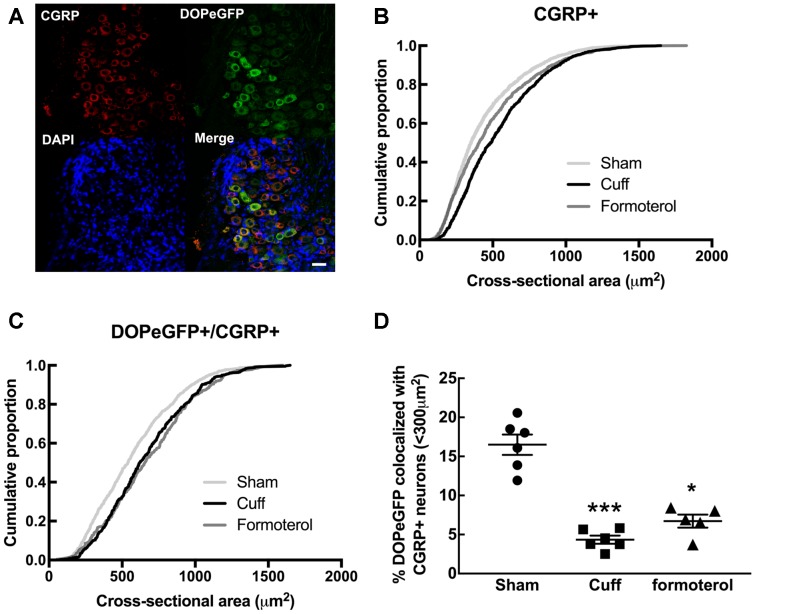
Impact of formoterol treatment on DOPeGFP colocalization with CGRP+ populations. **(A)** Representative fluorescence micrographs showing DOPeGFP co-localization with the neuronal marker calcitonin gene-related peptide (CGRP; red) as indicated by arrows in the overlay figure. Nuclei are stained with DAPI (blue). Scale bar 10 μm. **(B)** Distribution of CGRP+ neuronal populations in Sham (3,351 neurons, *n* = 7 animals; light gray), Cuff (1,331 neurons, *n* = 6 animals; black), and Formoterol (1,311 neurons, *n* = 5 animals; dark gray). **(C)** Distribution of neuronal populations co-expressing DOPeGFP and CGRP in Sham (803 neurons, *n* = 7 animals; light gray), Cuff (438 neurons, *n* = 6 animals; black) and Formoterol (294 neurons, *n* = 5 animals; dark gray) groups. **(D)** Percentage of cells co-expressing DOPeGFP and CGRP in neurons with areas <300 μm^2^ in Sham (*n* = 6; ●), Cuff (*n* = 6; ▪) or Formoterol (*n* = 5; ▴) animals. Values expressed as mean ± SEM. Kruskal–Wallis test and Dunn’s pos*t*-test: *p < 0.05, ***p < 0.001 vs. Sham.

Since DOPeGFP expression was decreased in unmyelinated peptidergic populations in Cuff mice (Figure 3) and (Ceredig et al., 2018), we assessed the consequence of chronic formoterol treatment on the global CGRP+ population and on the proportion of the CGRP+ population also expressing DOPeGFP (Figure 4). The cumulative distribution corresponding to CGRP+ neurons showed that the shift towards larger cell size values in the Cuff group is no longer present for the smallest cross-sectional areas after treatment with formoterol although the overall distribution remained significantly distinct from the Sham group (KS test: *D* = 0.090945, *p* = 3.398 10^−7^; Figure 4B). However, the cumulative distribution of the CGRP+DOPeGFP+ neurons remained similar to the cuff condition and was significantly different from the Sham condition (KS test: *D* = 0.15672, *p* < 2.2 10^−16^; Figure 4C) with the proportion of small size neurons (<300 μm^2^; 6.7 ± 0.8%) remaining similar to the Cuff condition (4.3 ± 0.5%) and lower compared to the Sham condition (16.5 ± 1.3%; KW test: *p* < 0.0001, Dunn’s *post hoc* test Sham vs. Cuff *p* = 0.0007, Sham vs. Formoterol *p* = 0.0352, Cuff vs. Formoterol *p* > 0.9999; Figure 4D). Therefore, chronic treatment with formoterol did not appear to restore the loss of DOPeGFP+ neurons induced by the neuropathic condition in small peptidergic neurons.

### DOPeGFP Expression at the Plasma Membrane in Formoterol-Treated Mice

**Figure 5:**
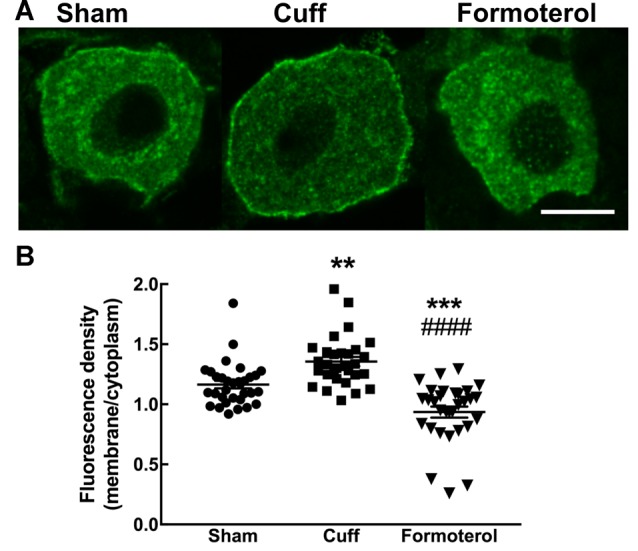
DOPeGFP expression at the cell surface in Formoterol-treated mice. **(A)** Representative fluorescence micrographs of DOPeGFP-positive neuron in Sham, Cuff and cuff animals treated with formoterol. Scale bar 10 μm. **(B)** DOPeGFP subcellular distribution was increased in neuropathic conditions and reduced after formoterol treatment compared to Sham. Data are expressed as means ± SEM (Sham: *n* = 32 cells from four animals, Cuff: *n* = 30 cells from three animals, Formoterol: *n* = 30 cells from three animals). One-way ANOVA and Tukey HSD *post hoc* test: **p < 0.01, ***p < 0.001 vs. Sham and ^####^ *p* < 0.0001 vs. Cuff, *p* > 0.5 Formoterol vs. Cuff.

Enhanced DOP expression at the plasma membrane was previously described in neuropathic conditions (Gendron et al., 2015). This observation was confirmed in the cuff model by showing that the ratio of fluorescence associated with the cell surface was significantly increased compared to the fluorescence associated with the intracellular compartments in the DOPeGFP+ neurons (Figure 5) as also previously reported (Ceredig et al., 2018). Formoterol treatment decreased membrane-associated fluorescence to values which were even lower than those of neurons in the Sham condition (Sham 1.16 ± 0.03, Cuff: 1.35 ± 0.04, formoterol 0.94 ± 0.05, one-way ANOVA *F*_(2,89_ = 28.8 *p* < 0.0001 Tukey HSD *post hoc* test Cuff vs. Sham *p* = 0.002, Cuff vs. Formoterol *p* < 0.0001, Sham vs. Formoterol *p* = 0.0002; Figure 5B). This indicates that treatment with formoterol suppressed the increase in DOP receptor surface expression observed in neuropathic mice.

### CGRP and DOPeGFP Expression in the Skin of Formoterol Treated Mice

**Figure 6:**
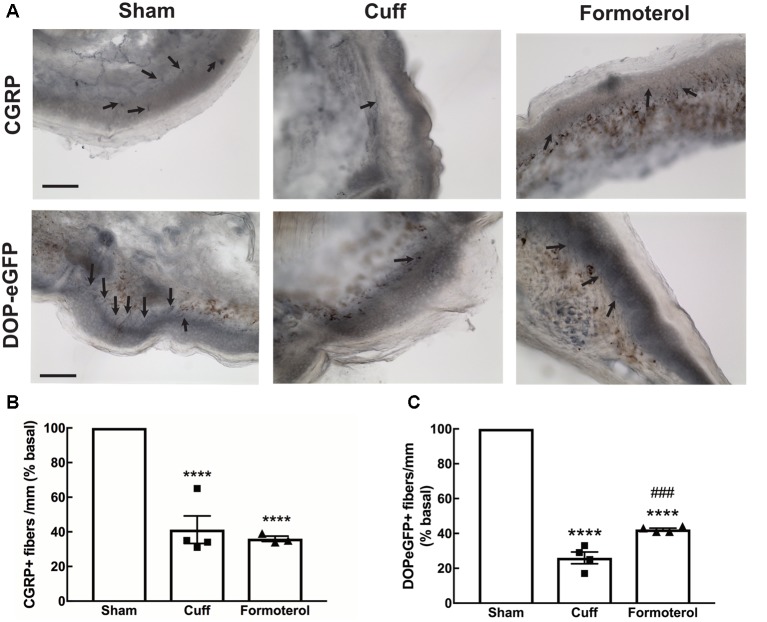
Density of CGRP+ and DOPeGFP+ free nerve endings in the skin. Representative micrograph of **(A)** CGRP or DOPeGFP intra-epidermal nerve fiber (IENF) labeling in the skin of Sham, Cuff and cuff animals treated with formoterol (black arrows). Scale bar 10 μm. **(B)** The density of CGRP+ free nerve endings in the glabrous skin of the right hind paw was decreased in Cuff animals and Cuff animals treated with formoterol. Data are expressed as means ± SEM, *n* = 4 mice for Sham and Cuff and *n* = 3 for Formoterol. One-way ANOVA and Tukey HSD *post hoc* test: ****p < 0.0001 vs. Sham. **(C)** The density of DOPeGFP+ free nerve endings in the glabrous skin of the right hind paw was decreased in Cuff animals and only partially restored in Cuff animals treated with formoterol compared to Sham. Data are expressed as means ± SEM, *n* = 4 mice per condition. One-way ANOVA and Tukey HSD *post hoc* test: ****p < 0.0001 vs. Sham, ^###^ *p* < 0.001 vs. Cuff.

A decrease in CGRP+ intra-epidermal nerve fiber (IENF) fiber length and density, as well as decrease in DOPeGFP+ IENF density, was reported 8 weeks post cuff surgery (Nascimento et al., 2015; Ceredig et al., 2018). We thus assessed whether treatment with formoterol impacted CGRP+ and DOPeGFP+ IENFs (Figure 6). The density of CGRP+ IENFs in the glabrous skin of the hind paw of cuffed mice treated with formoterol remained at a level comparable to the Cuff group (one-way ANOVA *F*_(2,8)_ = 49.15, *p* < 0.0001; Tukey HSD *post hoc* test Sham vs. Cuff *p* < 0.0001, Sham vs. Formoterol *p* < 0.0001, Cuff vs. Formoterol *p* = 0.771; Figure 6B). The density of DOPeGFP+ IENFs was higher in formoterol treated animals compared to the Cuff group but remained significantly lower compared to the Sham condition [one-way ANOVA *F*_(2,9)_ = 371, *p* < 0.0001; Tukey HSD *post hoc* test Sham vs. Cuff *p* < 0.0001, Sham vs. Formoterol *p* < 0.0001, Cuff vs. Formoterol *p* = 0.0008 (Figure 6C)]. As a whole, chronic formoterol treatment did not restore CGRP+ expression and induced partial recovery of DOPeGFP expression in the IENFs.

## Discussion

We first showed that oral administration of the β2 adrenergic agonist formoterol was as effective as its intraperitoneal injection (Yalcin et al., 2010) to alleviate mechanical allodynia in the cuff model of neuropathy. We then identified peripheral DOP receptors as mandatory for the antiallodynic effect and determined the impact of chronic formoterol on the expression of DOP receptors in the lumbar DRGs and skin IENFs.

Several studies pointed to the role of DOP receptors, and more specifically peripheral DOP receptors present in Nav1.8+ neurons, to counteract mechanical allodynia in neuropathic conditions induced by sciatic nerve ligation (Nadal et al., 2006; Gaveriaux-Ruff et al., 2011; Nozaki et al., 2012). Previous work by the group established that the antiallodynic action of chronic tricyclic antidepressant and SNRI treatments require DOP and β2 adrenergic receptors (Yalcin et al., 2010; Choucair-Jaafar et al., 2014; Kremer et al., 2018). Systemic administration of the DOP antagonist naltrindole also blocked the antiallodynic action of chronic administration of β2 adrenergic agonists (Yalcin et al., 2010; Choucair-Jaafar et al., 2014). In the case of the SNRI duloxetine, peripheral DOP receptors expressed in Nav1.8+ neurons were mandatory for the antiallodynic effect as no recovery was observed in the DOP cKO mouse line in which peripheral DOP receptors were selectively ablated in neurons expressing the Nav1.8 sodium channel (Ceredig et al., 2018). Here, we showed that peripheral DOP receptors expressed in Nav1.8+ neurons were also mandatory for the β2-adrenergic agonist formoterol to alleviate mechanical allodynia. Our results, therefore, establish that peripheral DOP receptors, likely located on C nociceptors, are necessary for the effective antiallodynic effect of the two chronic treatments. They also suggest that DOP receptor expression is modulated by the noradrenergic component of SNRIs.

In our previous work, we have characterized changes in DRG neuronal populations in the neuropathic condition resulting from sciatic nerve cuffing (Ceredig et al., 2018). Our main findings highlighted a decrease in the proportion of small size peptidergic neurons (≤300 μm^2^) and IENFs expressing DOPeGFP 8 weeks after cuff surgery as well as increased DOPeGFP expression at the plasma membrane (Ceredig et al., 2018), suggesting that these changes may contribute to control mechanical nociception. Here, we showed that chronic formoterol administration promoted partial recovery of DOPeGFP expression in free nerve endings in the skin but not in small CGRP+ neurons in the DRGs. This contrasts with the higher DOPeGFP expression found in small unmyelinated peptidergic neurons (≤300 μm^2^) following chronic duloxetine administration. The reason for this difference is unknown but it could correspond to the use of a suboptimal dose of formoterol as also suggested by the dose-response curve performed using intraperitoneal injections (Yalcin et al., 2010). It is indeed unlikely to depend on the serotoninergic component of the SNRI action since selective serotonin reuptake inhibitors did not alleviate mechanical allodynia (Benbouzid et al., 2008a). Our results, however, confirmed that DOP expression in the nerve terminals, likely unmyelinated peripheral axons of C mechanonociceptors (Brederson and Honda, 2015) appeared a primary determinant of mechanical sensitivity and might constitute a valuable marker of the neuropathic state.

Chronic pain was shown to increase DOP receptor translocation to the plasma membrane in the DRGs (reviewed in Gendron et al., 2015) and our data showed a similar increase in DOPeGFP surface expression in the cuff model. Chronic formoterol was associated with low DOP receptor expression at the plasma membrane that was even below values observed in the sham condition. The low DOPeGFP expression at the surface was also observed in mice chronically treated with duloxetine (Ceredig et al., 2018) and, therefore, seems a common mechanism by which the two antiallodynic treatments counteract mechanical hypersensitivity. The underlying mechanisms, however, remain elusive. DOP and β2 adrenergic receptors have been proposed to form heteromers based on studies performed in co-transfected cells (Jordan et al., 2001). However, β2 adrenergic receptors are expressed in satellite cells in the DRGs (Bohren et al., 2013) whereas DOP dependent analgesia is mediated at the neuronal level (Gaveriaux-Ruff et al., 2011; Ceredig et al., 2018; this work) and is independent of microglial activation (Mika et al., 2014) which precludes direct molecular interactions. The delayed antiallodynic action of both formoterol and duloxetine suggests that it requires cellular adaptations to take place. Lesion of peripheral noradrenergic fibers with guanethidine, a toxin that does not cross the blood brain barrier abolished the antiallodynic action of duloxetine (Kremer et al., 2018) supporting common involvement of peripheral β2 adrenergic receptors. Formoterol (Bohren et al., 2013) as well as antidepressants (Kremer et al., 2018) counteracted the increase in TNFα associated with neuropathic pain and downregulated the activity of the glial NFκB-TNFα pathway, a key regulator of proinflammatory cytokine production (Leung and Cahill, 2010). However, this anti-neuroinflammatory action is also shared by gabapentinoids that do not need opioid receptors for their antiallodynic action (Kremer et al., 2016b). This rather supports a view in which the anti-neuroinflammatory effect and the modulation of DOP expression and activity are not directly related.

There are few clues as to which DOP-dependent mechanisms mediate the anti-allodynic action following formoterol or SNRI chronic treatment. Current data point to an indirect effect through increased endogenous opioid peptide release by the native and adaptative immune systems, which can activate neuronal opioid receptors to alleviate mechanical allodynia (Binder et al., 2004; Celik et al., 2016). Indeed, noradrenergic sprouting consequent to nerve injury would activate the β2-adrenoceptors expressed by immune cells and promote the release of enkephalin, dynorphin and β-endorphin by these cells (Binder et al., 2004; Celik et al., 2016; Pannell et al., 2016). Similarly, enkephalins are also present in the skin (Slominski et al., 2011). Moreover, sympathetic fiber sprouting is known to take place in the skin in the cuff model (Nascimento et al., 2015) and is located close to cells expressing β2 adrenergic receptors and β-endorphin in inflamed paw tissue providing a way to activate not only MOP but also DOP receptors (Binder et al., 2004). Activation of DOP receptors could, in turn, induce pain relief by reducing Nav1.8 channel activity *via* inhibition of p38 mitogen-activated protein kinase as described for Nav1.7 channels in a rat model of diabetic neuropathy (Chattopadhyay et al., 2008).

In summary, our work established that DOP receptors expressed in Nav1.8+ neurons were mandatory for the anti-allodynic action of chronic treatment with the β2 adrenergic agonist formoterol. It also revealed that chronic formoterol partially reversed the loss of peripheral DOP receptors in the skin and counteracted enhanced DOP expression at the plasma membrane. Our study thus adds to current literature pointing to potential interest in repositioning β2 adrenergic agonists for the treatment of neuropathic pain.

## Data Availability Statement

Images and raw data will be available upon request to the corresponding author.

## Ethics Statement

All experiments were approved by the “Comité d’Ethique en Matière d’Expérimentation Animale de Strasbourg” [authorization number 201503041113547 (APAFIS#300).02] and conducted in agreement with the EU Directive 2010/63/EU for animal experiments.

## Author Contributions

RC, IY, CG-R, MB, ES, and DM designed experiments. RC, FP, SD, UA, PH, IY, and DM performed experiments. RC, UA, FP, and DM analyzed the data. RC, IY, CG-R, MB, and DM wrote the manuscript.

## Conflict of Interest

The authors declare that the research was conducted in the absence of any commercial or financial relationships that could be construed as a potential conflict of interest.

## Abbreviations

ANOVA: analysis of variance
CGRP: calcitonin gene-related peptide
cKO: conditional knockout
DOP: delta-opioid
DRG: dorsal root ganglion
eGFP: enhanced green fluorescent protein
IENF: intraepidermal nerve fiber
KS: Kolmogorov–Smirnov
KW: Kruskal–Wallis
PWT: paw withdrawal threshold
SNRI: serotonin noradrenaline reuptake inhibitor.
